# A new method for evaluating the impacts of semantic similarity measures on the annotation of gene sets

**DOI:** 10.1371/journal.pone.0208037

**Published:** 2018-11-27

**Authors:** Aarón Ayllón-Benítez, Fleur Mougin, Julien Allali, Rodolphe Thiébaut, Patricia Thébault

**Affiliations:** 1 Univ. Bordeaux, Inserm UMR 1219, Bordeaux Population Health Research Center, team ERIAS, Bordeaux, France; 2 Univ. Bordeaux, CNRS UMR 5800, LaBRI, Bordeaux, France; 3 Univ. Bordeaux, Inserm UMR 1219, INRIA SISTM, Bordeaux, France; 4 CHU de Bordeaux, Pole de sante publique, Service d’information medicale, Bordeaux, France; 5 Vaccine Research Institute, Creteil, France; King Abdullah University of Science and Technology, SAUDI ARABIA

## Abstract

**Motivation:**

The recent revolution in new sequencing technologies, as a part of the continuous process of adopting new innovative protocols has strongly impacted the interpretation of relations between phenotype and genotype. Thus, understanding the resulting gene sets has become a bottleneck that needs to be addressed. Automatic methods have been proposed to facilitate the interpretation of gene sets. While statistical functional enrichment analyses are currently well known, they tend to focus on well-known genes and to ignore new information from less-studied genes. To address such issues, applying semantic similarity measures is logical if the knowledge source used to annotate the gene sets is hierarchically structured. In this work, we propose a new method for analyzing the impact of different semantic similarity measures on gene set annotations.

**Results:**

We evaluated the impact of each measure by taking into consideration the two following features that correspond to relevant criteria for a “good” synthetic gene set annotation: (i) the number of annotation terms has to be drastically reduced and the representative terms must be retained while annotating the gene set, and (ii) the number of genes described by the selected terms should be as large as possible. Thus, we analyzed nine semantic similarity measures to identify the best possible compromise between both features while maintaining a sufficient level of details. Using Gene Ontology to annotate the gene sets, we obtained better results with node-based measures that use the terms’ characteristics than with measures based on edges that link the terms. The annotation of the gene sets achieved with the node-based measures did not exhibit major differences regardless of the characteristics of terms used.

## 1 Introduction

The recent revolution in new sequencing technologies, as a part of the continuous process of adopting new innovative protocols [1] has strongly impacted our understanding of the relations between phenotype and genotype. In addition to commonly used gene-by-gene analyses in which the transcription of hundreds of specific genes is quantified by polymerase chain reaction in each study, gene set approaches are becoming increasingly popular. Currently, the studies consider groups of genes that are often related as they co-occur in the same pathway [2]. Several genes in a given pathway that are slightly differentially expressed are more relevant than only one gene with a strongly different expression pattern.

This new field of research has become unavoidable over the last two decades and is based on the inference of gene sets according to a comparison of experimental results under diverse conditions. In such context, sets of genes that contribute to specific phenotypes are inferred based on experimental results obtained under various experimental conditions. For example, Chaussabel and Baldwin [3] used experimental conditions related to various diseases to decipher the genes that may be involved in the innate or specific immune response. An additional issue involves the interpretation of these gene sets using the information available for each gene, which is based on annotation terms derived from a wide range of sources. These terms provide functional information for each gene with varying levels of details according to the current state of knowledge in the research area. At the gene set level, the joint interpretation of such information involves gathering and synthesizing the contents of numerous terms. The computing process of these terms has become crucial, as reported in burgeoning scientific studies using and developing methods dedicated to gene set annotations over the last two decades [4].

One popular approach used to interpret biological information related to gene sets is based on enrichment statistical methods (for a review, see [4]). These methods aim to retain the over-represented terms (*i.e.*, overused by the genes in the gene set) without considering the relevance of the information and specificity of these terms. Thus, the results supplied by enrichment tools consist of lists of numerous over-represented terms, and a postprocessing stage remains necessary to remove the potentially redundant information. Therefore, DAVID [5] uses an *a posteriori* analysis of the annotation terms co-utilized by the genes to cluster the genes into potential groups with similar information. However, manual expertise still remains crucial and becomes unachievable if the number of gene sets to be analyzed is too high. Moreover, these methods provide redundant information by selecting terms that are hierarchically related, leading to difficulty and occasional bias in correctly interpreting the results. To address these issues, other initiatives using the similarities among the over-represented terms have been proposed. For example, Revigo [6] is a postprocessing tool that selects only some terms from the output results of enrichment methods.

Another solution, called GO SLIM annotation, involves severely reducing the number of terms that are used for the annotation *a priori*. Thus, ten customized GO SLIM annotations currently maintained by the Gene Ontology Consortium mainly focus on various subgroups of organisms (*e.g.*, plant, yeast). While many uses of SLIM annotation have been proposed for specific projects, only a few attempts have been made to automatically compute them [7]. Davis *et al.* [7] used both graph theory and information theory to calculate the optimal reduced GO graph while penalizing graph complexity. Jin and Lu [8] used the frequency of annotation terms to identify an informative subset of GO terms that maintains the maximum semantic information. In contrast, Na *et al.* [9] mapped GO terms from gene sets to GO SLIM terms interpreted as functional categories of interest according to domain experts. Such SLIM mapping approaches can also be combined with enrichment methods to reduce the number of over-represented terms.

However, defining the functional categories of interest at the beginning of the analysis is not an easy task and requires prior knowledge of the scope of interest. In this context, this research is motivated by our belief that the reduction in the number of terms should be performed *a priori* by considering the whole set of terms in a gene set before using any enrichment method. Then, the main task relies on the clustering of annotation terms according to their proximity by computing the *semantic similarity* among these annotation terms. This notion is used to compare two entities belonging to one or two knowledge resources. The idea is not to compare the two entities exclusively in relation to their wording but rather to consider their semantic context (*i.e.*, all features related to the two entities to be compared).

Thus, semantic similarity measures provide a solution for the complementary or redundant information related to highlighted genes in a gene set of interest. This strategy unavoidably facilitates the biological interpretation of a gene set that may contain hundreds of genes. Given the significant number of published semantic similarity measures [10–12], the selection of a metric can be tricky. A recent publication discussed this issue and proposed a classification of these measures according to the type of driven analysis [13].

Nevertheless, to the best of our knowledge, no real work has been proposed to evaluate the impact of using a given semantic similarity measure over another measure. A relevant gene set annotation needs to meet specific features to provide relevant information to domain experts. We propose to evaluate the impact of each measure while considering the following features that define the relevant criteria for a “good” synthetic gene set annotation [14]:

- The number of annotation terms has to be drastically reduced, while the relevant terms that representatively annotate the gene set must be retained (designated as **synthesis**).
- The number of genes described by the selected terms is designated as **coverage**.

Finding the best possible compromise between both features is not an easy task while attempting to maintain a **sufficient level of details** supplied by the selected terms. In this context, we propose a new pipeline to evaluate the impact of semantic similarity measures in annotating gene sets. To illustrate and discuss the discrepancies among these measures, we use Gene Ontology to annotate the genes and analyze the results according to the two research works that generated the gene sets (modules) for deciphering the immune response [3, 15].

## 2 Materials

We first present the Gene Ontology and then introduce the features of the terms that can be used by semantic similarity measures. Finally, we detail the measures chosen for our investigation based on the features they use.

### 2.1 Gene ontology

Gene Ontology (GO) was designed to describe the roles of genes in any biological organism [16]. GO is organized according to three distinct categories, also known as ontologies, namely, Molecular Function, Cellular Component, and Biological Process. GO contains more than 44,000 terms connected through different kinds of relations: hierarchical (namely *is_a* and *part_of*) and transversal (*e.g.*, *regulates* and more recently *starts_with*). As the relations between terms are oriented, GO ontologies are directed acyclic graphs (DAGs). Of all GO terms, nearly 11,000 terms are molecular functions, nearly 4,000 terms are cellular components, and nearly 30,000 terms are biological processes.

GO is the most popular knowledge source used by the biology community to annotate genes. The GO Annotation (GOA) database has been developed for this purpose, specifically to associate GO terms to a gene in a given organism to describe its biological roles [17].

As this study is performed using data related to humans, the following numbers are based on *GOA human*, which is the GOA file listing GO terms used to annotate human genes. Overall, to describe 19,575 human genes (of over 23,000—http://www.ensembl.org/Homo_sapiens/Info/Annotation), 280,711 distinct (gene, GO term) pairs exist regardless of the evidence code (and after eliminating annotations with *No Data* evidence code and annotations with the *NOT* qualifier). This process results in an average of 14.3 terms per human gene, implying that the interpretation of a set containing 100 genes may require the processing of 1400 GO terms. Obviously, this cannot be performed manually, and computational methods are required for this task.

### 2.2 Semantic similarity measures

#### 2.2.1 Features of GO terms

Each GO term has many features that can be used to establish its similarity with other GO terms, including its depth (*i.e.*, the longest path from the root of the ontology to the term—the *root* being Biological Process, Cellular Component or Molecular Function), the set of its descendants (*i.e.*, its child terms and recursively until the leaves), the set of its ancestors (*i.e.*, its parent terms and recursively until the root) and its information content (also referred as IC). The following two kinds of IC metrics exist: extrinsic and intrinsic.

The extrinsic category was the first introduced and the corresponding metrics used external knowledge. The most famous metric in this category is the metric introduced by Resnik in 1995 [18], which is based on the frequency of a concept within a corpus. In the context of GO, this IC is related to the occurrence of a GO term within GOA as follows: the more frequently a term is used to annotate genes, the lower its IC value. Its IC (IC_R_) is defined as follows:

$$IC_{R}\left(T\right)=-log\left(p\left(T\right)\right)$$ (1)

where *p(T)* is the ratio of the number of genes in a given organism annotated by term *T* to the total number of annotations within the GOA file of this organism. Subsequently, Seco *et al.* [19] argued that the IC should be computed independently from external knowledge. More precisely, these authors claimed that the IC should be intrinsic and that only knowledge contained within the ontology should be used to compute such a measure. Their IC (IC_S_) is defined as follows:

$$IC_{S}\left(T\right)=1-\frac{log(|descendants(T)|+1)}{log(|descendants(Root)|)}$$ (2)

where *descendants* is the set of descendants of the term *T* within GO. However, as emphasized by Mazandu and Mulder [20], this type of IC considers all descendants similarly regardless of their depth. Thus, this approach does not distinguish terms with different specificities. To address this issue, other intrinsic ICs have been proposed [21]. In particular, Mazandu and Mulder introduced an alternative intrinsic IC [20] that considers the depth of *T* and its descendants, as follows:

$$IC_{GOu}\left(T\right)=-ln\left(p\left(T\right)\right)$$ (3)

where

$$p\left(x\right)=\{\begin{array}{cc} 1 & \text{if}\;x\;\text{is}\;\text{a}\;\text{root}.\\ \prod_{t\in ancestors(x)}\frac{p(t)}{\left|descendants(t)\right|} & \text{otherwise}. \end{array}$$ (4)

Furthermore, the following features of the two GO terms to be compared may also be considered in quantifying their similarity: their distance (*i.e.*, the shortest path from one GO term to the other GO term) and their common ancestors. Concerning the latter, the following two main features have been introduced for distinguishing ancestors potentially more relevant than others: (i) the lowest common ancestor (LCA) which is the most specific, *i.e.*, having the maximal depth, and (ii) the most informative common ancestor (MICA) which is the common ancestor with the highest IC value.

#### 2.2.2 Investigated semantic similarity measures

Pesquita *et al.* proposed a survey of semantic similarity measures to compare the terms in the context of GO [10]. Authors have categorized these measures according to the following three classes: (i) “node-based”, which is based on the features of the GO terms, (ii) “edge-based”, which is based on the number of edges that exist between two GO terms, and (iii) hybrid, which is based on a mix of the methods used in the two previous classes. More recently, Guzzi *et al.* conducted a survey of existing semantic similarity measures [11]. The authors displayed the latter using a Venn diagram, which facilitates the identification of the features used by many measures and also those that are used by only a few measures. In addition, measures that harness multiple features can be easily identified at the intersection of multiple ovals. Mazandu *et al.* recently realized an additional review of semantic similarity measures (referred to in their paper as “term semantic similarity”) [12]. These authors provided an exhaustive list of the different ICs that have been proposed in the literature and refined the classification proposed by Pesquita *et al.* by adding the following subcategory to the node-based measures: graph-based measures. This subcategory has been previously introduced by Mazandu and Mulder [21] for specifying measures based on the ancestors and/or descendants of the terms to be compared. The authors emphasized that the old measures only used the MICA or simply counted the number of descendants of the terms, which is more limited. The purpose of this paper is not to propose a new categorization of existing semantic similarity measures for comparing GO terms but rather to evaluate their varying impact while analyzing gene sets. Thus, we selected nine pairwise semantic similarity measures according to the classifications provided by Pesquita *et al.* [10], Guzzi *et al.* [11] and Mazandu *et al.* [12] by choosing at least one measure belonging to each category. The measures are listed above with a description of the feature(s) that they use.

- Ganesan [22] adapted to the comparison of terms by Sanfilippo *et al.* [23]: the longest path from the root to both terms and their LCA;
- Leacock & Chodorow [24] normalized (LC): the shortest path between the two terms and the maximal depth within the ontology;
- Pekar & Staab (PS) [25]: the shortest path between the two terms and the shortest path between their LCA and the root term;
- Zhou [26]: the IC_S_ of each term and of their MICA as well as the shortest path between the two terms and the maximal depth within the ontology;
- Resnik [18] normalized according to Jain and Bader’s approach [27]: the IC_R_ of the MICA of the two terms and the maximal value of the IC_R_ within GO;
- Lin [28]: the IC_R_ of each term and of their MICA;
- Nunivers [20]: the IC_GOu_ of each term as well as the IC_GOu_ of their MICA;
- Distance Function (DF) [29]: the ancestors of the two terms;
- Aggregate IC (AIC) [30]: the IC_R_ of ancestors of the two terms.

**Fig 1:**
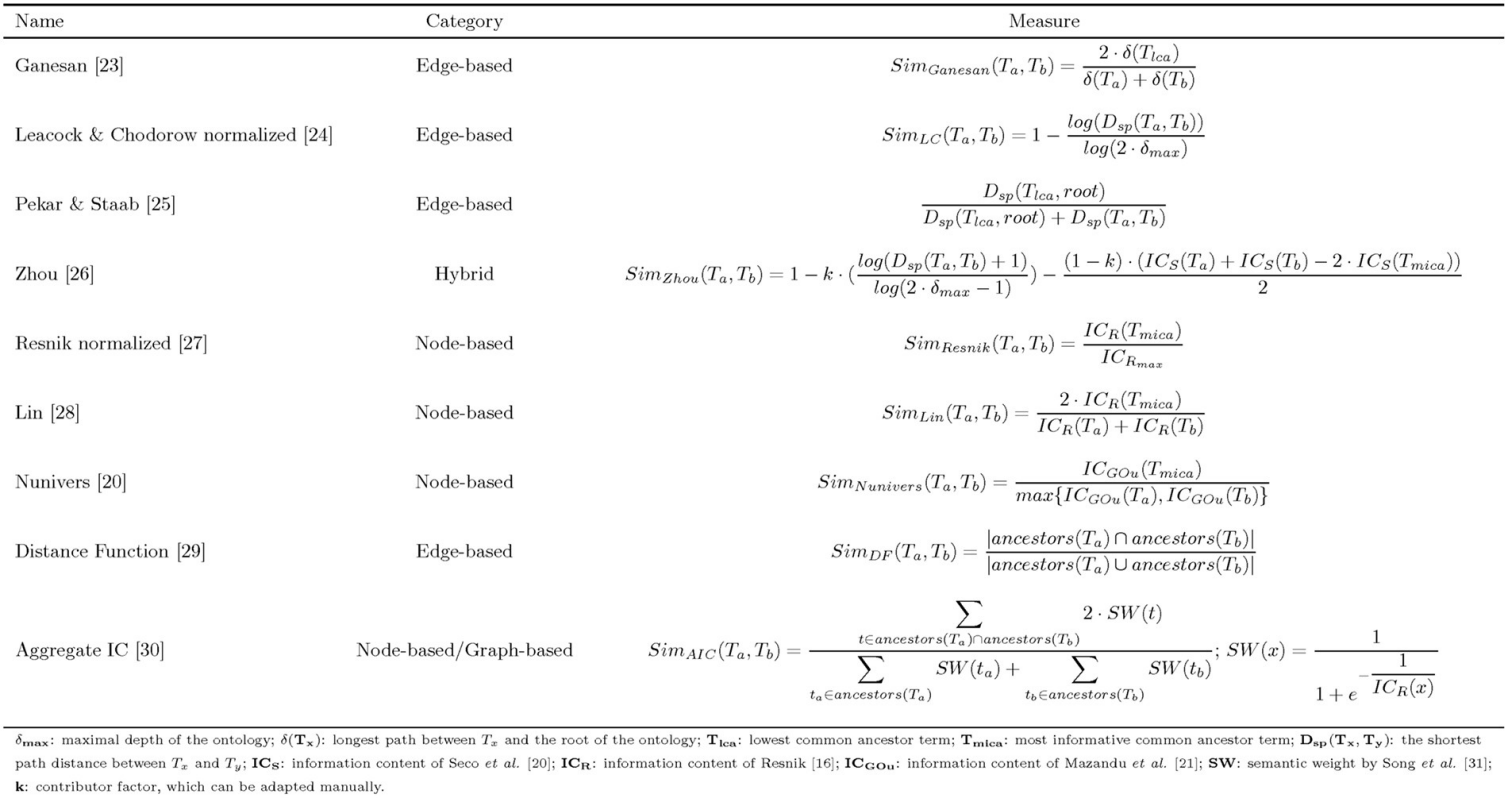
Nine semantic similarity measures investigated in this study.

**Fig 2:**
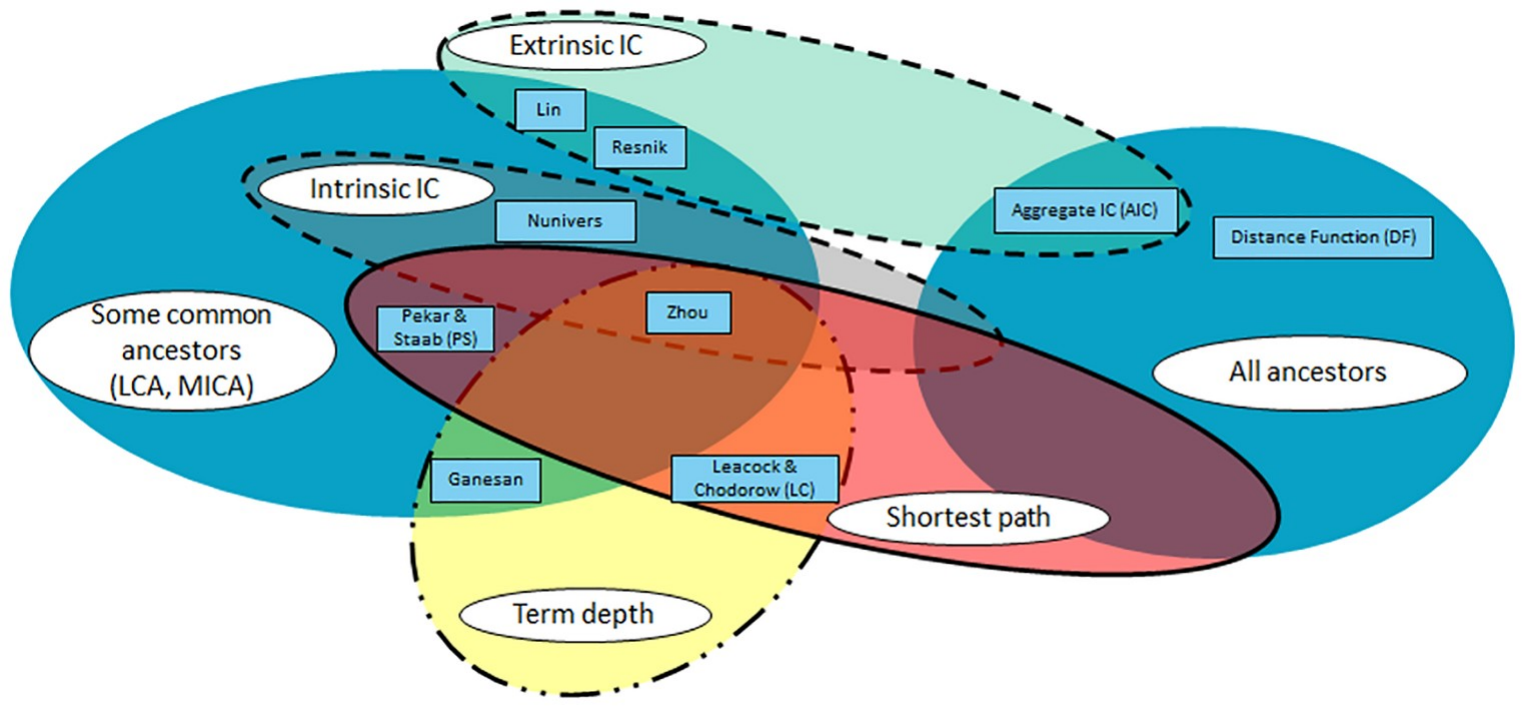
Classification of the semantic similarity measures investigated in this study according to the features that they use (adaptation from [11]).

As shown in Fig 1, we chose three edge-based measures, five node-based measures (including a graph-based measure) and one hybrid measure. We selected more node-based measures than edge-based measures because the latter were deemed less efficient in comparing GO terms [10]. Fig 2 is adapted from the categorization proposed by Guzzi *et al.* [11] to illustrate that the measures investigated in this study use all of the usual features that were described in the previous subsection. To consider the two previously introduced categories of IC, we replaced the “Term IC” feature used by Guzzi *et al.* [11] with the following two distinct features: “Extrinsic IC” (*i.e.*, the IC_R_) and “Intrinsic IC” (*i.e.*, the IC_S_ and IC_GOu_). Notably, we did not choose any vector space model-based measure as these measures compare terms not only according to features specific to the terms within the ontology but also according to external knowledge (*i.e.*, the genes they annotate).

## 3 Methods

We describe the workflow implemented to study the impacts of different semantic similarity measures while interpreting gene sets. First, we eliminated redundancy and incompleteness among the GO annotation terms before computing the semantic similarity matrices of each pair of terms. Second, we assessed the ability of each semantic similarity measure to compute the best partitions of the annotation terms by evaluating the clustering partition fitness and estimating the impact of varying hierarchical clustering methods. Finally, we examined the effectiveness of each semantic similarity measure in (i) reducing the number of annotation terms while selecting the most representative terms of the gene set and (ii) providing annotation for the maximum number of genes included in the set. The combination of these two criteria, which are mostly quantitative, allowed for the estimation of the ability of each semantic similarity measure to yield a more relevant and synthetic annotation of gene sets as previously described.

### 3.1 Elimination of redundant and incomplete annotations

The first stage of reduction was easily applied by eliminating redundancy [31] among the GO terms that annotate each gene set. This stage consists of eliminating annotations that involve a GO term if at least one of its descendant terms annotate the same gene.

The second stage aimed to eliminate incomplete annotations. According to the definition given by Faria *et al.* [32], incompleteness corresponds to cases where the function of a gene is not fully described. Among the existing GO annotations, authors have emphasized that GO terms that are too generic constitute incomplete annotation. Thus, we decided to remove such GO terms. To identify these incomplete terms, we used an alternative measure to that proposed by Faria *et al.* [32], who considered GO terms with more than 10 descendants as incomplete annotations. More precisely, we computed the IC_GOu_ distribution of the GO terms used in GOA human. Then, we removed the GO terms whose IC_GOu_ was in the first quartile.

### 3.2 Clustering of annotation terms computed semantic similarity matrices

**Fig 3:**
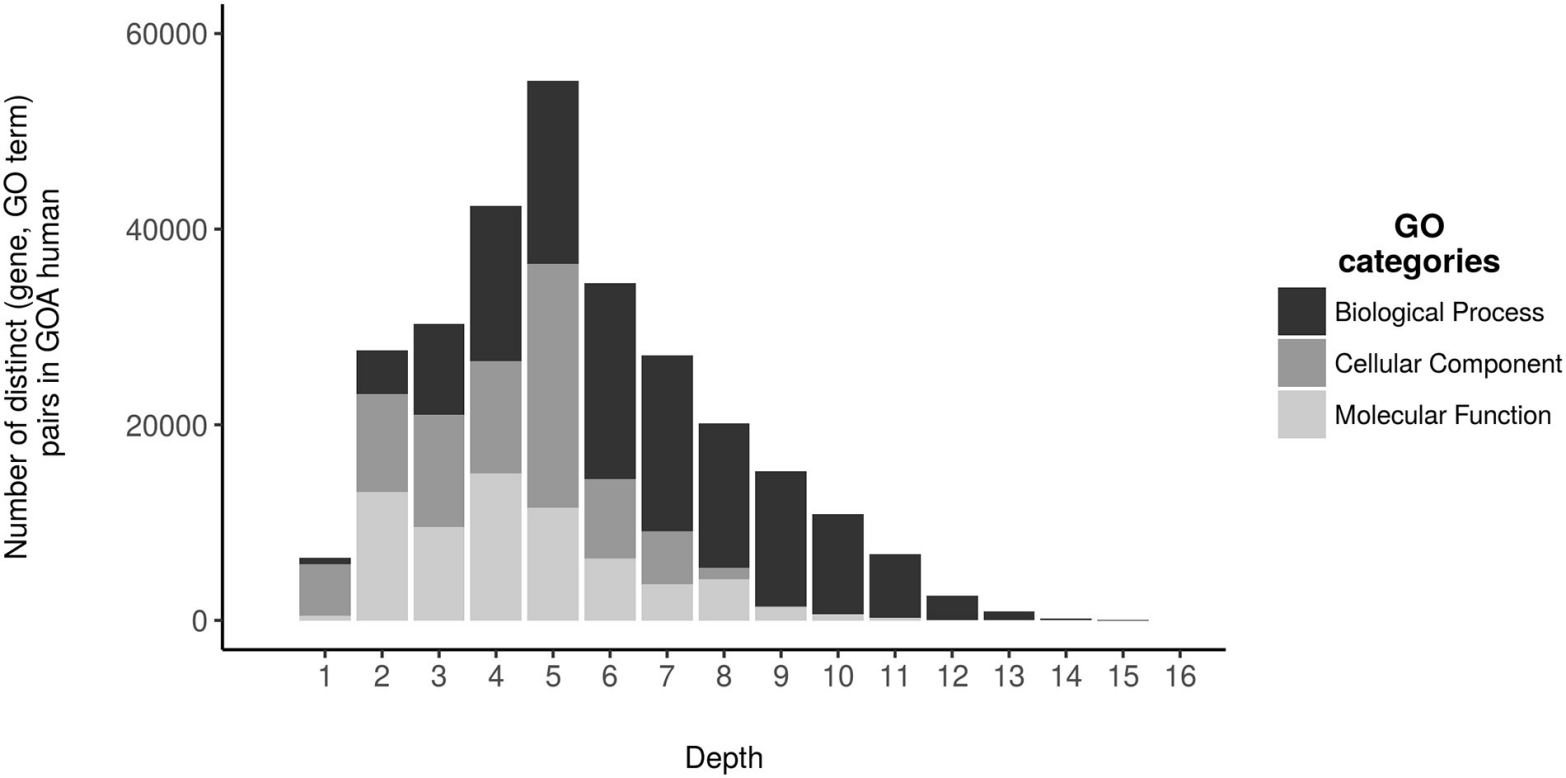
Depth of GO terms in the annotations provided by GOA human.

According to Thomas [33], biological processes represent the objectives that an organism is “programmed” to realize. In addition, most specific annotation terms (*i.e.*, with a depth over 5) belong to the Biological Process ontology (Fig 3). Based on these considerations, we reduced the scope of our analysis by strictly focusing on the GO terms in this ontology since they provide a clear interpretation of the roles of genes.

For each of the nine measures displayed in Fig 1, we computed matrices containing the semantic similarity value of each pair of GO terms, corresponding to biological processes that are a part of the investigated gene sets (described in detail in subsection 4.1). Second, we used agglomerative hierarchical clustering methods to compute clusters of terms [34]. The choice of using unsupervised hierarchical clustering methods was motivated by the hierarchical relations that connect the annotation terms represented within a DAG [35]. Using the list of pertinent features provided by Hennig [36], the following three characteristics are required to define “good” clusters: (i) the within-cluster dissimilarity has to be small, (ii) the between-cluster dissimilarity has to be large, and (iii) the terms in a cluster have to be well-represented by the centroid or a very small group of terms (to help the identification of synthetic and relevant terms).

The most classical hierarchical clustering methods differ in how they define the perimeter of the resulting clusters when adding a new term. In the single linkage hierarchical method (*SLHM*), the distance between two clusters is given by the shortest distance that can be calculated between two terms from these two clusters. In the complete linkage hierarchical method (*CLHM*), this distance corresponds to the longest distance between two terms. Finally, the average linkage hierarchical method (*ALHM*) uses the average distance between each term from each cluster.

#### 3.2.1 Clustering partition fitness

Even if no relation exist among the data, hierarchical clustering techniques always create partitions. Consequently, assessing the relevance of the resulting partitions is essential. To test the ability of the clustering approaches to provide good fits to the data, we computed the cophenetic correlation coefficient (*CCC*) [37] of the resulting dendrograms. This metric aims to measure how the original pairwise distance between terms (given by semantic similarity measures) is retained within the computed dendrogram. The CCC score between original term pairwise distance matrix *Y* and dendrogram *Z* is calculated as follows:

$$CCC\left(Y,Z\right)=\frac{\sum_{i<j}\left(Y_{ij}-y\right)\left(Z_{ij}-z\right)}{\sqrt{\sum_{i<j}{\left(Y_{ij}-y\right)}^{2}\sum_{i<j}{\left(Z_{ij}-z\right)}^{2}}}$$ (5)

where *Y*_*ij*_ is the distance between terms *i* and *j* given by the original pairwise matrix *Y*, and *Z*_*ij*_ is the distance between *i* and *j* computed within dendrogram *Z*. The *y* and *z* values refer to the average distances within *Y* and *Z*, respectively.

Thus, we used the three clustering methods mentioned above while varying the semantic similarity measures. This score aims to compare the distances between the data (i) within the dendrogram and (ii) within the original matrix. The resulting coefficient corresponds to the square of the coefficient of determination, denoted *r*^2^, and indicates the proportion of variance explained by the clustering results. Thus, a value close to 1 reflects a perfect correspondence.

#### 3.2.2 Variations induced by the clustering methods

In this section, we did not use the *SLHM* method because of its ineffectiveness in clustering data showing hierarchical relations (as illustrated using the investigated gene sets in subsection 4.2.1).

Based on the assumption that the best semantic similarity measure should be able to reproduce the same term partition while varying the clustering methods, we compared the partitions given by *CLHM* and *ALHM* using the *Z-index* [38]. This value gives a criterion for evaluating the differences between two clustering methods for a fixed semantic similarity measure. This *Z-index* is computed as follows:

$$Z=\sum_{k}Z_{k}=\sum_{k}\sum_{i}\frac{|x_{1ijk}-x_{2ijk}|}{|x_{1ijk}\left|+\right|x_{2ijk}|}$$ (6)

where *x*_1*ijk*_ and *x*_2*ijk*_ indicate whether a pair *ij* of terms are within the same cluster in dendrograms *X*_1_ and *X*_2_. All potential partitions are simultaneously considered by varying k. The *Z* value provides the distance between two clustering methods by computing the difference in the status (*i.e.*, whether both terms are in the same cluster) of each pair at each stage of the procedure (by varying the *k* value).

Thus, the smaller the differences between the results provided by both clustering methods, the more robust the semantic similarity measure regardless of the clustering methods. This measure assesses the possible combinations of all pairs of terms, which could be interpreted as a comparison of the intermediate structures given by both dendrograms before obtaining the different partitions (without considering a given *K* number of optimal partitions *a priori*). Then, this criterion can be considered a generalization of the classical metrics used to compare two partitions [38] and provides a score ranging from 0 and 1 that correspond to a perfect resemblance and dissemblance between the two dendrograms generated by each clustering method.

Subsequently, we concentrated on the hierarchical clustering method, which exhibited the best results according to the *CCC* score. Using an unsupervised clustering method, which is a classical approach, we determined the optimal number of clusters based on the computation of the average silhouette width (*ASW*) score [34]. This method uses the geometrical measures of cluster compactness and separation [39], which are calculated while varying the number of clusters, *i.e.*, cutting the dendrogram at different levels. For a given number K of clusters, we compute the average of the silhouette width. For each resulting cluster, the silhouette width is calculated as follows:

$$s_{i}=\frac{b_{i}-a_{i}}{\max\{a_{i},b_{i}\}}$$ (7)

where *i* is a given term within a cluster, *a*_*i*_ is the average distance from *i* to all other terms within its cluster, and *b*_*i*_ is the minimum average distance from *i* to all other terms in any other cluster. Thus, the average silhouette width (ASW) of the clusters for a given *K* is given by:

$$ASW_{K}=\frac{\sum_{i\in n}s_{i}}{n}$$ (8)

where *n* refers to the total number of terms.

Then, considering the results obtained for each set of *K* clusters, the optimal partition (*i.e.*, the *K* value) can be deduced from the highest *ASW* score. The *ASW* score is then computed from the two sets of modules to analyze its distribution. To guide the interpretation of the *ASW* score distributions, a score below 0.25 or above 0.50 might correspond to an artificial or real structure within the data [40]).

#### 3.2.3 Variations induced by the semantic similarity measures

To provide a meaningful analysis, we assessed the efficiency of the semantic similarity measures in infering synthetic and relevant terms using the resulting partitions. Identifying the best trade-off between summarizing the information and preserving a good level of precision (given by the depth of terms) was challenging. Thus, first, only some terms were selected for each cluster, and second, the resulting clusters that annotated only a few genes were not included. Then, we compared the semantic similarity measures based on their capacity to provide: (i) a synthetic annotation with relevant terms and (ii) a good coverage of the gene set while finally guaranteeing a fine-grained annotation.

**A**—**Identifying and filtering representative terms**

The clustering method generates clusters of annotation terms that exhibit a certain level of similarity. The purpose of this step was to identify the most synthetic and pertinent GO terms, which are subsequently designated as **representative terms**, of each cluster to summarize their annotation information. The algorithm proposed for identifying such representative terms is provided in the next sub-section. Only representative terms that annotate a minimum of three genes in the investigated gene sets were retained, designated as **synthetic terms** throughout the remainder of the article. We applied this filter because our aim was to obtain a synthetic annotation at the end of the proposed pipeline.

**Description of the algorithm to identify representative terms**

To identify the representative terms of a cluster, we developed a new algorithm based on the functions MSRT (Most Specific Representative Terms) and FCT (Find Combined Terms). All terms are represented by a bitset that is set to the size of the cluster and initialized to all zeros. Bitsets are used to compare the terms while traversing the ontology. Each position of a bitset is associated to a term present in the original cluster, and this position of each bitsets that are associated to the ancestor terms of this term is set to 1. Then, the bitset associated to a term summarizes if this term is hierarchically related to some terms of the cluster.

The MSRT algorithm tries to identify for a given top term (initially set up to Biological Process) a set of terms more specific in their biological meaning. Thus, the related bitsets are compared. If the bitsets are equal, the top term can be ignored and the corresponding descendant terms are retained as candidate representative terms.

Then, the FCT algorithm is called for each candidate representative term returned by MSRT. As MSRT, the FCT algorithm compares bitsets to identify a combination of more specific terms related to the same terms of the cluster.

At the end, we retain the combination(s) of representative terms having the best *IC* value. The *IC* of a combination is given by the mean *IC* of *ICs* of each term of the combination.

**Algorithm 1**: MSRT(*top*_*term*, *GO*)

**Input**: *top*_*term* is a term,

   *GO* is an object with two attributes: (i) *bitset*(*x*) is the bitset associated to term x and (ii) *children*(*x*) is the list of terms that are children of x.

**Ouput**: *representative*_*terms* is the set of specific representative terms.

**1** *terms*_*to*_*visit* ≔ ∅; *representative*_*terms* ≔ ∅; *visited*_*terms* ≔ ∅

**2** push(*terms*_*to*_*visit*, *top*_*term*)

**3 while** *terms*_*to*_*visit* ≠ ∅ **do**

**4**  *candidate*_*term* ≔ pop(*terms*_*to*_*visit*)

**5**  **if** *candidate*_*term* ∉ *visited*_*terms* **then**

**6**   *is*_*child*_*representative* ≔ *false*

**7**   push(*visited*_*terms*, *candidate*_*term*)

**8**   **for** *child*_*term* ∈ *GO*.*children*(*candidate*_*term*) **do**

**9**    **if** *GO*.*bitset*(*child*_*term*) ⩵ *GO*.*bitset*(*top*_*term*) **then**

**10**     *is*_*child*_*representative* ≔ *true*

**11**     push(*terms*_*to*_*visit*, *child*_*term*)

**12**    **end**

**13**   **end**

**14**   **if** *is*_*child*_*representative* ⩵ *false* **then**

**15**    push(*representative*_*terms*, *candidate*_*term*)

**16**   **end**

**17**  **end**

**18 end**

**19 return** *representative*_*terms*

**Algorithm 2**: FCT(*representative*_*term*, *k*, *sct*, *GO*)

**Input**: *representative*_*term* is a term representing the cluster,

   *k* is the maximum number of combinations,

   *sct* is the minimum number of bits shared by terms,

   *GO* is an object with two attributes: (i) *bitset*(*x*) is the bitset associated to term x and (ii) *children*(*x*) is the list of terms that are children of x.

**Output**: *representative*_*terms*_*set* is a set of sets of most specific representative terms.

**1 if** *k* > *size*(*GO*.*children*(*representative*_*term*)) **then**

**2**  *k* ≔ *size*(*GO*.*children*(*representative*_*term*))

**3 end**

**4** *combinations*_*set* ← all combinations of *k* terms from *GO*.*children*(*representative*_*term*)

**5** *representative*_*combination*_*found* ≔ *false*

**6 for** *combination* ∈ *combinations*_*set* **do**

**7**  *GO*.*bitset*(*combination*) ← union of the bitsets of terms part of *combination*

**8**  *number*_*of*_*shared*_*bits* ← number of bits shared by terms part of *combination*

**9**  **if** *GO*.*bitset*(*combination*) ⩵ *GO*.*bitset*(*representative*_*term*) ***and***

**10**    *number*_*of*_*shared*_*bits* < *sct* **then**

**11**   *combined*_*representative*_*terms* = ∅

**12**   **for** *term*_*in*_*combination* ∈ *combination* **do**

**13**    push(*combined*_*representative*_*terms*, MSRT(*term*_*in*_*combination*, *GO*))

**14**   **end**

**15**   push(*representative*_*terms*_*set*, *combined*_*representative*_*terms*)

**16**   *representative*_*combination*_*found* ≔ *true*

**17**  **end**

**18 end**

**19 if** *representative*_*combination*_*found* ⩵ *false* **then**

**20**  push(*representative*_*terms*_*set*, {*representative*_*term*})

**21 end**

**22 return** *representative*_*terms*_*set*

**B**—**Evaluating the efficiency of the semantic similarity measures in synthesizing annotation terms**

**Fig 4:**
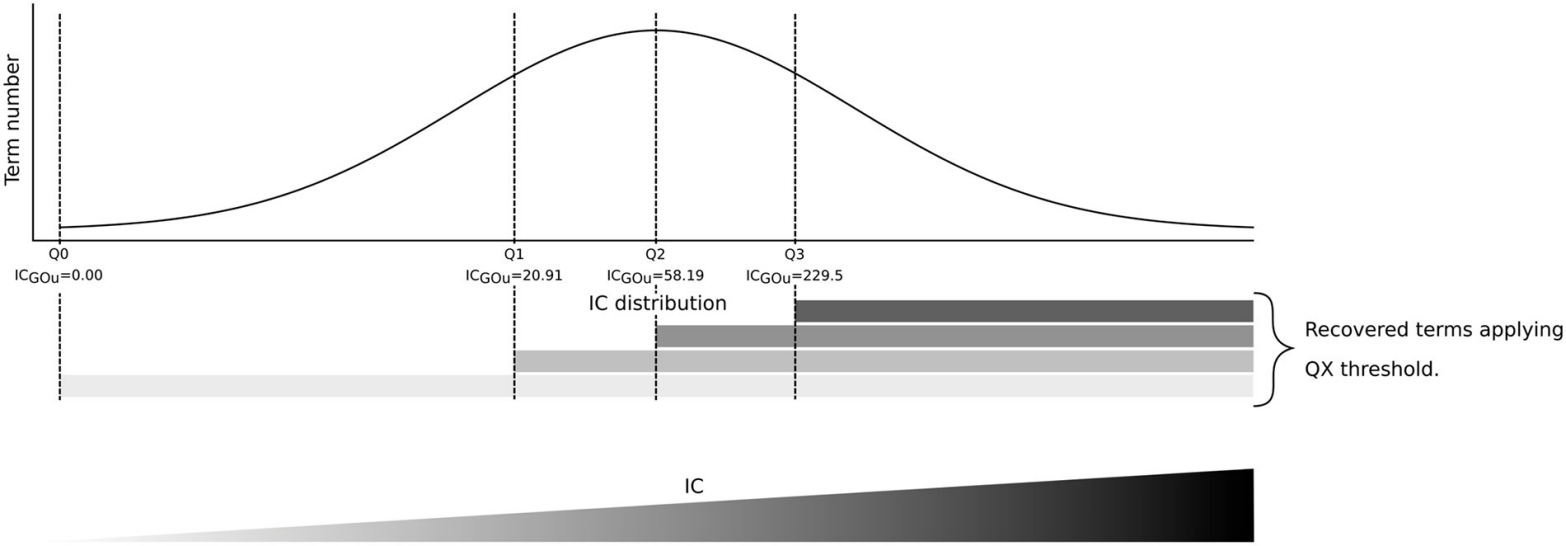
IC_GOu_ distribution of GO terms used in GOA human.

Then, we compared the impacts of the semantic similarity measures by examining the representative terms obtained by each measure. As previously stated in the introduction, a suitable synthetic gene set annotation reduces the number of terms while maintaining a sufficient level of details within the synthetic terms. Based on the distribution given by the computed IC_GOu_ values of the whole set of terms used in GOA human, we divided the value range into four contiguous intervals with an equal density of terms (Fig 4). This categorization of the results was used to assess the semantic similarity measures’ impacts on: (i) the number of synthetic terms selected to annotate the genes and (ii) their level of details. Furthermore, the distinction between two methods that equally decrease the number of terms or are scored with the same number of related genes has to be analyzed in depth according to the level of details given by each group of terms. In practice, the cumulative percentage of synthetic terms was computed for each quartile of the IC_GOu_ values by considering the term and gene coverage sides simultaneously.

## 4 Results

### 4.1 Experimental data

We concentrated our study on two human data experimental datasets to illustrate our analytic framework.

The *Chaussabel and Baldwin* dataset comprises 260 genes sets called modules that were computed according to their expression profile achieved from one to fifteen experimental protocols using a coexpressed network approach [3]. Each of these protocols corresponds to a study of a disease with the aim of identifying genes that trigger an immune response. More precisely, the modules that gather genes with a similar behavior within the fifteen experimental protocols may be related to the immune response in general. In contrast, modules of genes that show a similar behavior in a limited number of protocols may be expressed specifically in some diseases.

The *BTM* (blood transcriptional modules) dataset comprises 346 modules and aims to characterize innate and adaptive immune responses in vaccination studies [15]. The modules were built through large-scale data integration of publicly available transcriptomes of human blood using “interactome”, “bibliome”, pathway databases and specific biological contexts to deduce a set of transcription modules.

### 4.2 Clustering of annotation terms using computed semantic similarity matrices

#### 4.2.1 Clustering partition fitness

**Fig 5:**
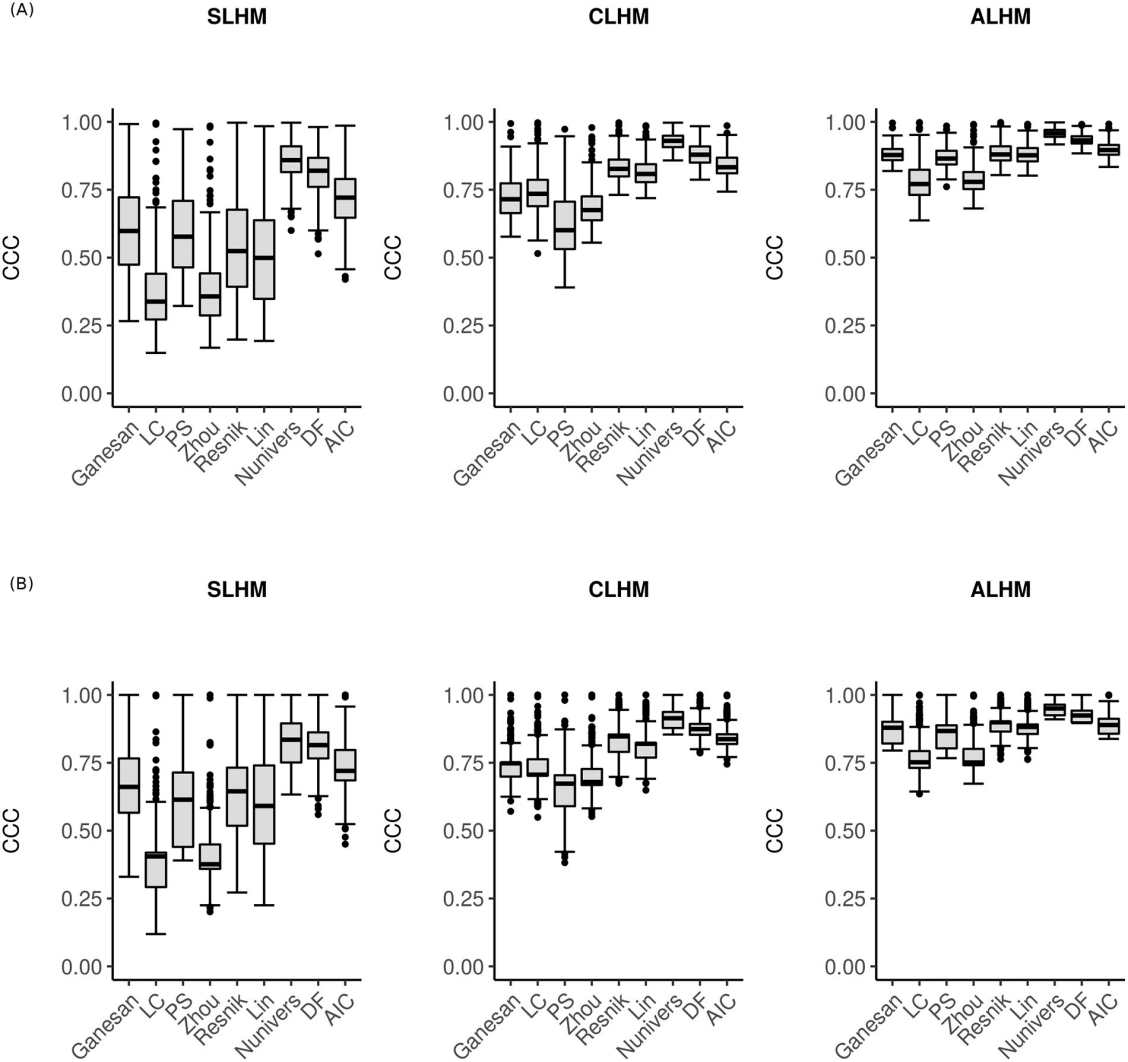
Cophenetic correlation coefficients (*CCC*) according to various semantic similarity measures of the investigated modules. (A) from Chaussabel and Baldwin and (B) BTM. The following three linkage hierarchical methods are presented: single (SLHM), complete (CLHM) and average (ALHM).

Each module in both datasets has been used to retrieve annotation terms related to the genes within the modules. Then, the semantic similarity between each pair of annotation terms was computed. To derive a quality score for the clusters of terms, we analyzed the *CCC* value. As shown in Fig 5, we observe that the *CCC* scores given by the *SLHM*, *CLHM* and *ALHM* methods follow the same trend as they tend to increase or decrease according to the semantic similarity measures. However, there is a significant difference in the *CCC* score dispersion among the clustering methods. We observe that the *CCC* scores are noticeably lower with the *SLHM* method (*e.g.*, the *CCC* median scores of the LC and Zhou measures are below 0.50). The observation of such low varying degrees of quality could be expected for *SLHM* because this clustering method is known to often perform consecutive additions of terms and is more often used to reveal gradients in a dataset. The dispersion of scores according to the modules and semantic similarity is less important for the other two clustering methods, and in both cases, the *CCC* median scores are the highest for the same measures, *i.e.*, DF and Nunivers. Notably, the Nunivers measure has the greatest *CCC* score with a very small dispersion according to the modules. In contrast, the measures that provide the most significant *CCC* score dispersion are not systematically the same according to *CLHM* and *ALHM*.

While the best clustering quality score was obtained by using the *ALHM* method, the following question emerges: for a given semantic similarity measure and a given module, do we retrieve the same partitions of terms regardless of the clustering method used? Thus, how similar are the partitions given by both clustering methods for a given module and a given semantic similarity measure? If the partitions are highly similar, this information could be consider reliability and robustness criteria for the semantic similarity measure due to the little influence of the clustering methods.

#### 4.2.2 Variations induced by the clustering methods

**Fig 6:**
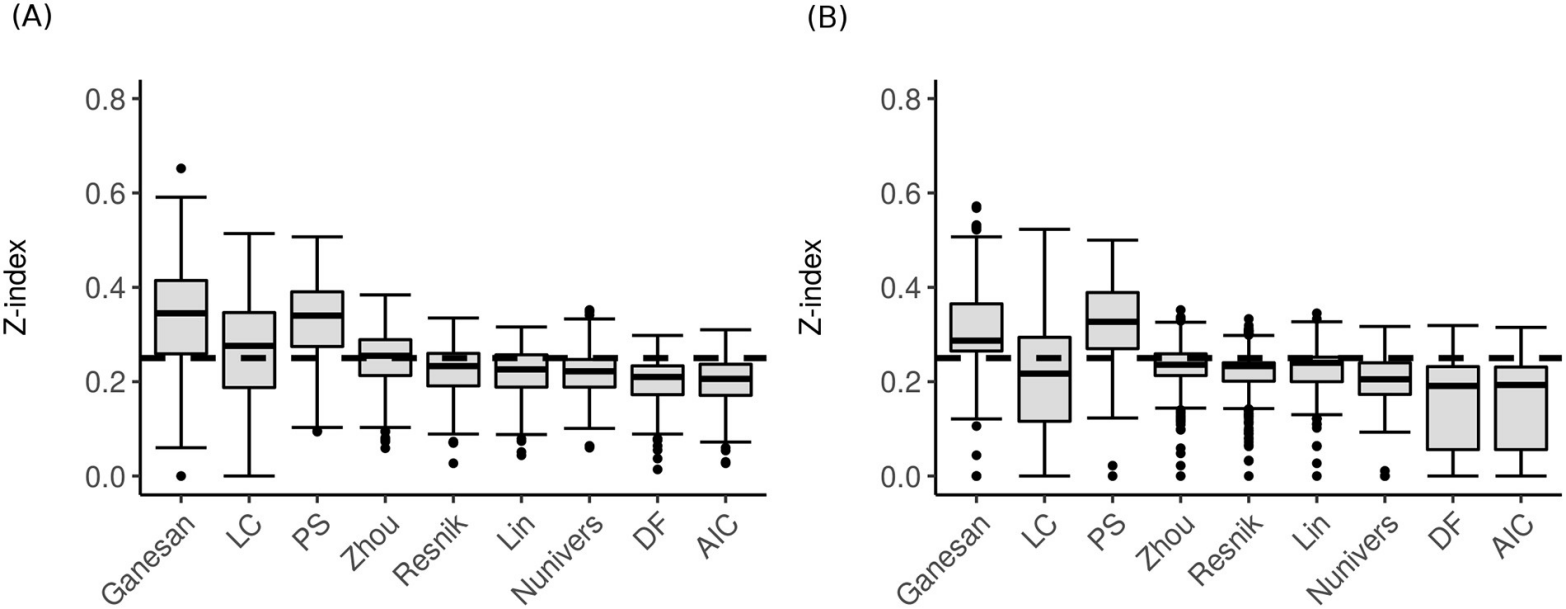
*Z-index* scores for comparing the whole structure of the dendrograms using *CLHM* and *ALHM* for the investigated modules. (A) from Chaussabel and Baldwin and (B) BTM.

We used the *Z-index* to compare and decipher the similarities and differences between both partitions given by the *CLHM* and *ALHM* methods (Fig 6). Based on the assumption that a pertinent semantic similarity measure should provide the same clusters regardless of the adopted clustering approach, we assume that the smaller the *Z-index* score, the greater the similarity between two distinct clustering method partitions. Thus, most semantic similarity measures give similar results with *Z-index* scores smaller than 0.25, except for the Ganesan and PS measures considering both datasets (LC and Zhou were also below 0.25 when computed using the Chaussabel and Baldwin dataset).

As an intermediate conclusion, based on this analysis, we can highlight five semantic similarity measures (LC, Zhou, Resnik, Lin, Nunivers, DF and AIC) for which the choice of *CLHM* or *ALHM* does not show a significant impact regardless of the dataset. However, clustering using *ALHM* provides better *CCC* scores (Fig 5) and was thus used for further analysis.

#### 4.2.3 Variations induced by the semantic similarity measures

Finally, we analyzed the impact of each semantic similarity measure according to the specific features that may be good markers providing relevant information to domain experts.

**A**—**Identifying and filtering representative terms**

**Fig 7:**
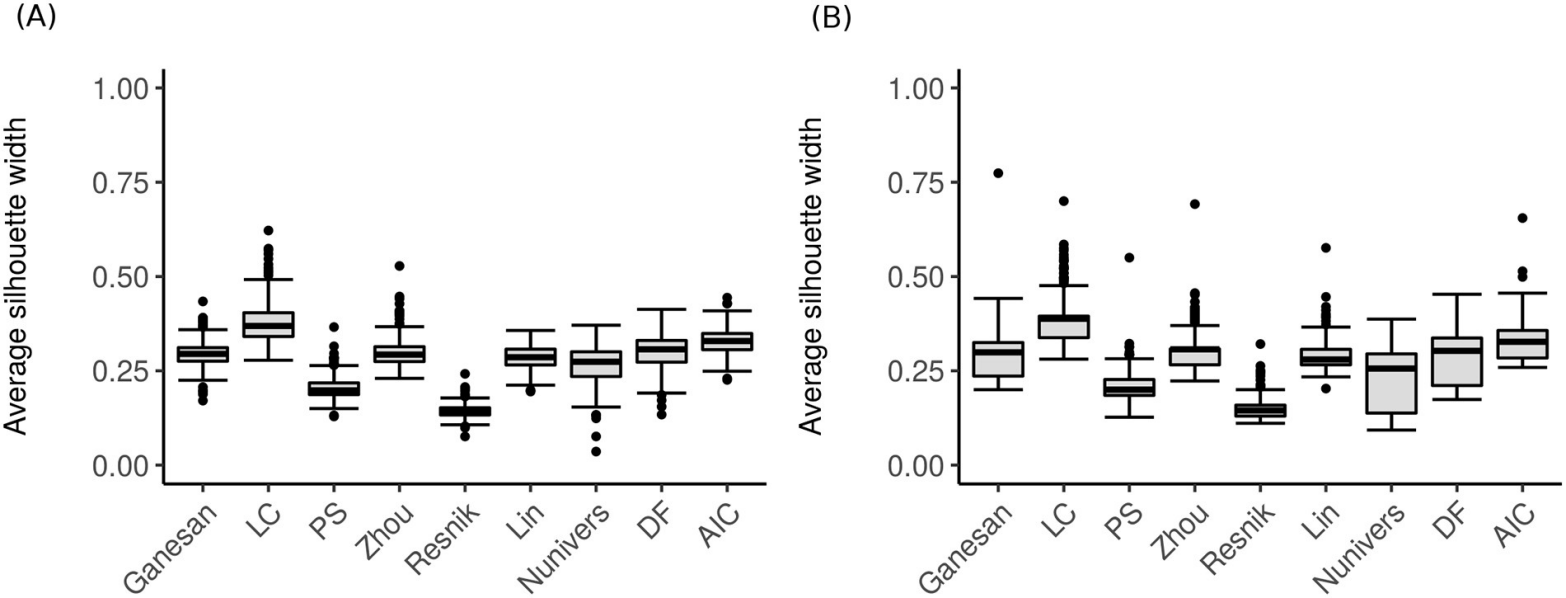
Average silhouette width (*ASW*) computed by *ALHM* according to each semantic similarity measure of the investigated modules. (A) from Chaussabel and Baldwin and (B) BTM.

First, after applying the representative algorithm, we analyzed the quality of the clusters of terms using the *ASW* score. This score was computed for each module in both datasets. As shown in Fig 7, we observe that the *ASW* score is below 0.25 for the PS and Resnik measures and between 0.26-0.50 for the other measures. Thus, no semantic similarity measure managed to compute a global partition with a structuring score above 0.50, which is the threshold used to separate the unstructured from structured clusters. This observation must be moderated by the specificity of the data in which an important number of genes are still unknown in some modules (as represented in S1 and S2 Figs, in which the coverage of the annotated genes can vary significantly depending on the modules) and, consequently, badly annotated [41, 42].

To obtain insight into the features given by the representative terms and based on the observations deduced from Fig 7, we applied a filtering step to concentrate our analysis on the terms that best uniformly describe the modules. Thus, we did not include representative terms related to fewer than three genes.

Considering the criteria used to define a “good” synthetic gene set annotation, we addressed the following key questions: what is the level of details given by the remaining representative terms? What is the number of related genes?

**B**—**Evaluating the efficiency of the semantic similarity measures in synthesizing annotation terms**

**Fig 8:**
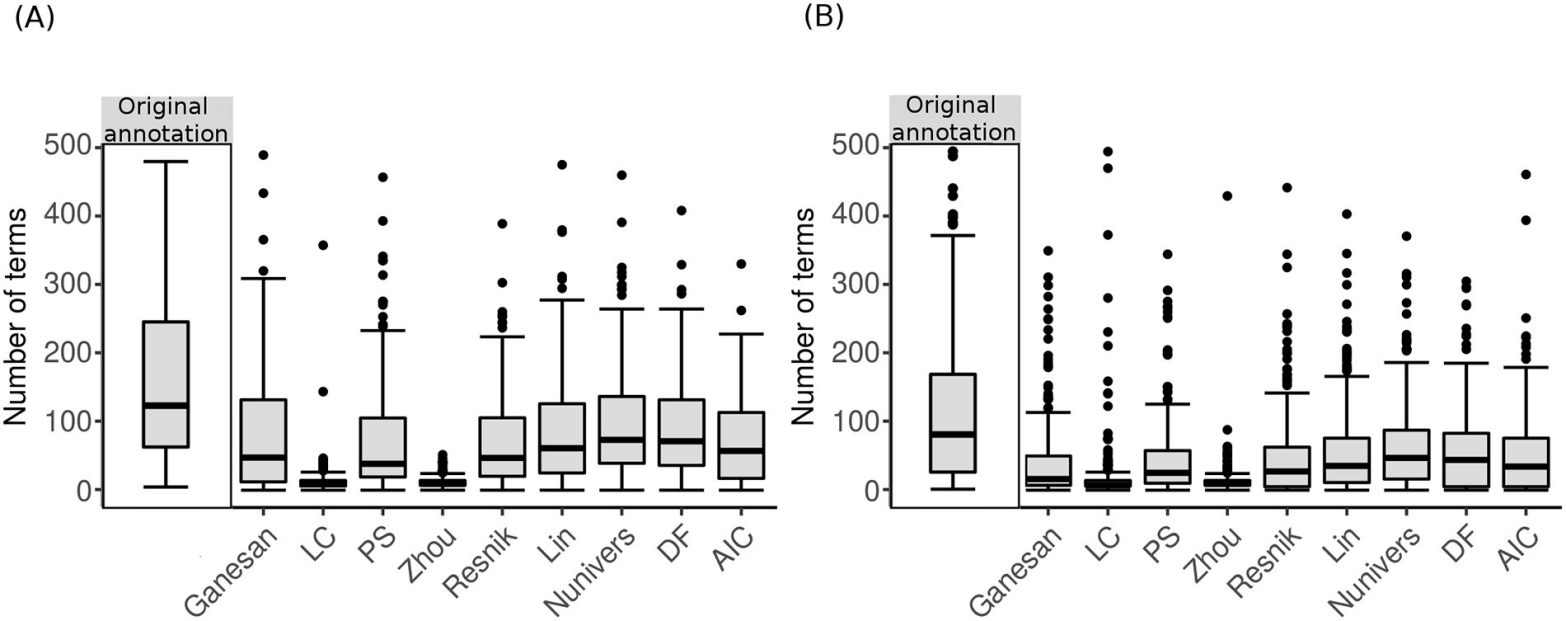
Number of representative terms obtained after applying each semantic similarity measure for the investigated modules. (A) from Chaussabel and Baldwin and (B) BTM. The “Original annotation” box-plot corresponds to the initial number of annotation terms.

To assess the impact induced by each semantic similarity measure, we first evaluated the reduction in the number of terms between the original set of annotation terms and the filtered representative terms. As shown in Fig 8, we observed a subsequent drastic decrease in the number of terms using all semantic similarity measures. In particular, the most striking decrease is observed in the LC and Zhou measures, where the remaining number of terms is very small.

**Fig 9:**
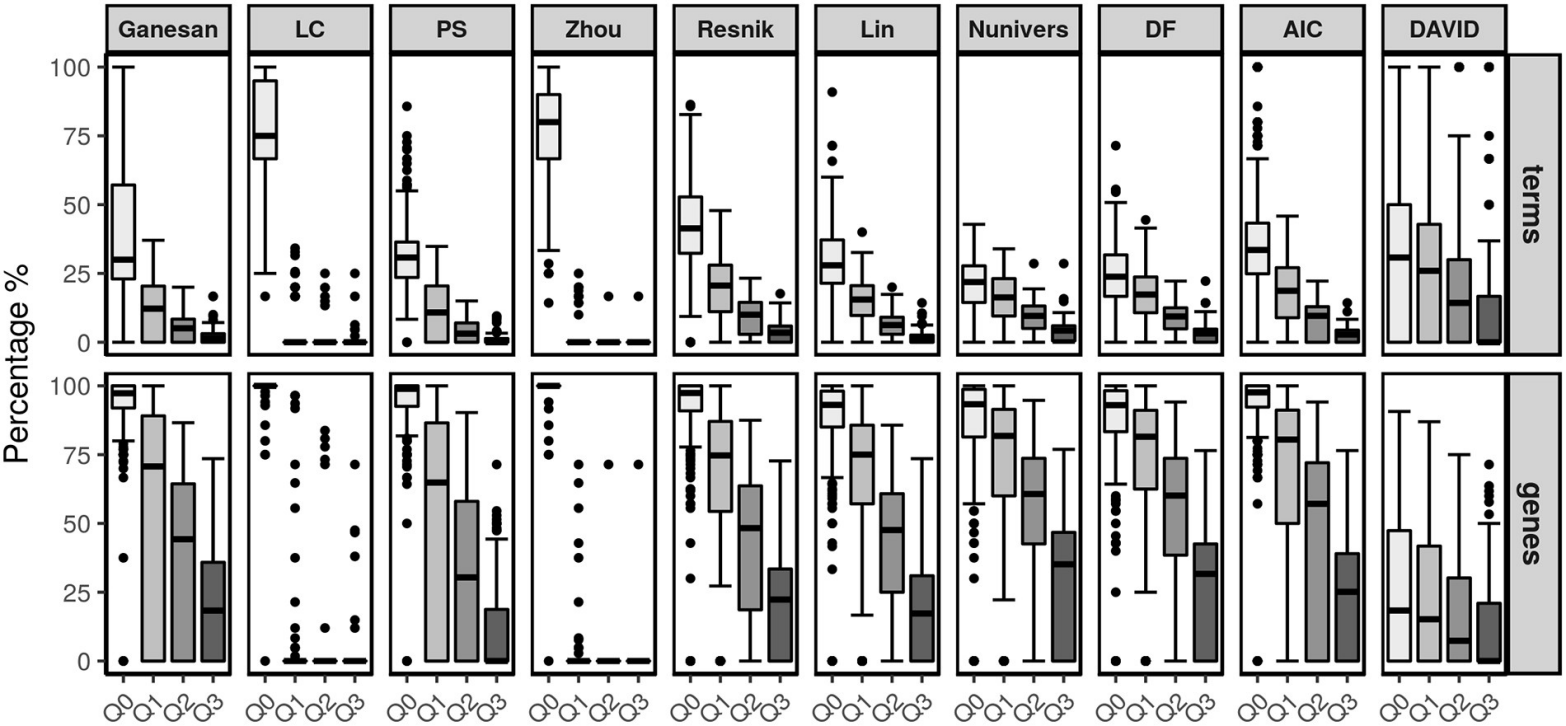
Percentage of synthetic terms and covered genes using each semantic similarity measure and comparison with the DAVID enrichment tool for Chaussabel and Baldwin’s modules.

**Fig 10:**
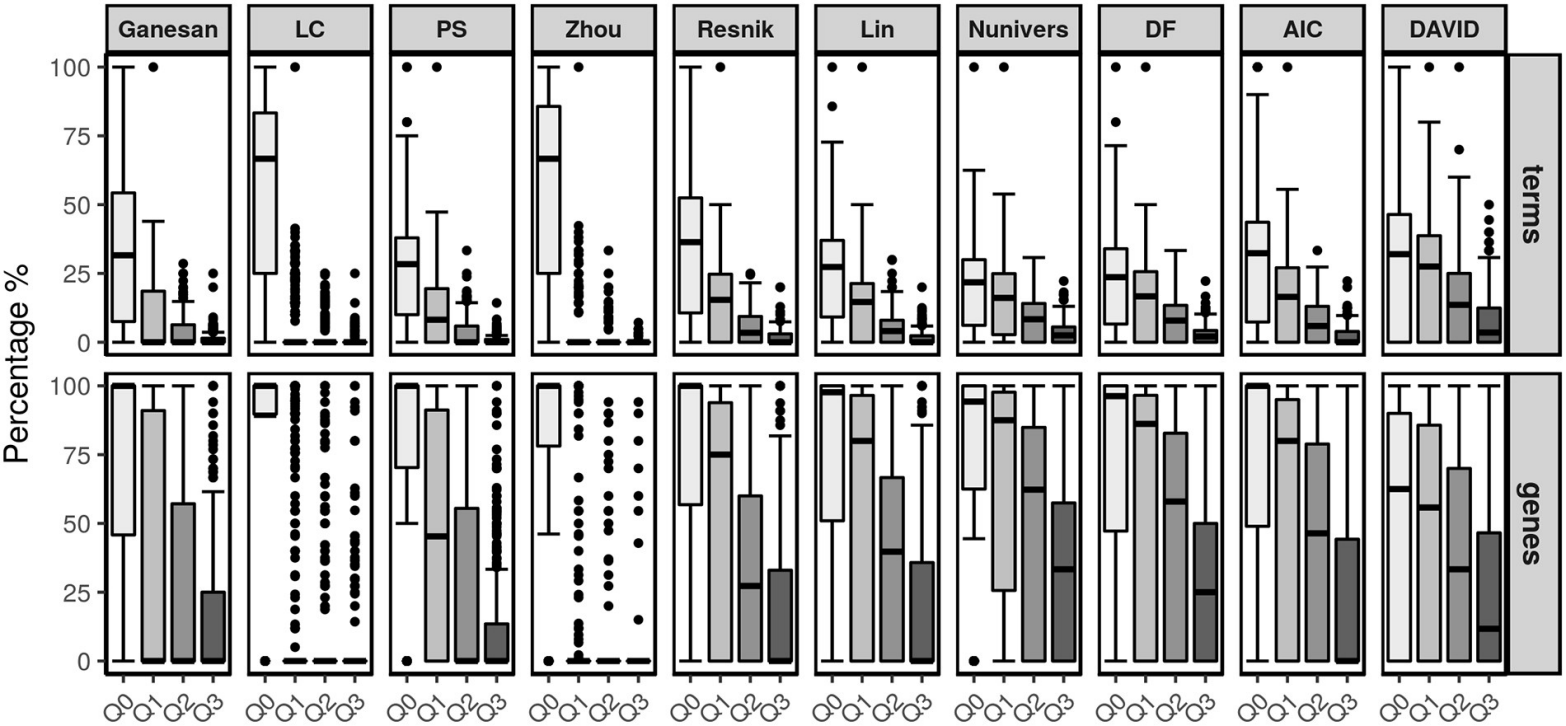
Percentage of synthetic terms and covered genes using each semantic similarity measure and comparison with the DAVID enrichment tool for the BTM.

The box-plots shown in Figs 9 and 10 display the ratio between the number of synthetic terms and the number of representative terms. For each measure, four box-plots are shown to qualitatively represent the percentage of synthetic terms according to the four categories based on the continuous interval of the IC_GOu_ scores. These percentages of terms were aggregated from right to left since the cumulative effect allows for the representation of the first quartile *Q*_0_, *i.e.*, the final percentage of the synthetic terms. As a reading guide, one may differentially consider the *Q*_0_ box-plot in which the global annotation view is represented and the three other box-plots in which the fine-tuned analysis is presented. Based on the terms, the relative analysis aims to qualify the best measures when their median line is low and, in contrast, based on the genes, when their median line is high.

The first line in each figure provides results focused on the terms. The downward trend observed in the first quartile *Q*_0_ is less marked for the LC and Zhou measures. For these measures, the filtering stage had a less noticeable effect as a larger number of synthetic terms was retained. Of particular interest, their respective box-plots given for the three other quartiles show that only a few terms with pertinent levels of information were retained. In contrast, Resnik, Nunivers, DF and AIC provided the best results with a larger number of synthetic terms having IC_GOu_ scores between the first and third quartiles regardless of the dataset. The other measures are more sensitive to the type of experimental data (*e.g.*, Ganesan shows interesting results for the Chaussabel and Baldwin dataset, whereas it performs poorly with the BTM). Finally, the same analysis was performed using the results given by the DAVID tool [5]. Thus, we applied the enrichment method and only retained the resulting terms that annotate at least three genes. The decrease in terms from *Q*_0_ to *Q*_3_ is less pronounced than all semantic similarity measures, corresponding to a higher number of more important terms with a high IC_GOu_ score.

The second line in each figure highlights the related gene coverage according to the same quartiles before and after applying the pipeline. The first observation considers the *Q*_0_ box-plots in which all semantic similarity measures nearly achieve a 100% coverage. However, in the other box-plots, the LC and Zhou measures show singular box-plot profiles compared to the other measures. Indeed, most genes are related to synthetic terms with the weakest IC_GOu_ scores (*i.e.*, within the first quartile of values). Thus, these two measures positively retained a small number of terms (from *Q*_1_ to *Q*_3_) with the disadvantage of a low level of details. In contrast, Resnik, Lin, Nunivers, DF and AIC have better IC_GOu_ scores considering the percentages of the *Q*_1_ to *Q*_3_ categories of terms regardless of the dataset. Among these measures, we focus on Nunivers and DF because most specific terms (within the last quartile of the IC_GOu_ score) still annotate more than 25% of the genes. From this perspective, the other measures appear to be sensitive to the type of dataset. Finally, considering DAVID, we observe that its coverage of genes is lower than that of most semantic similarity measures. However, notably, DAVID performs better with the BTM, while simultaneously, it does not provide a gene coverage above 60% for half of the modules.

## 5 Discussion

An important finding of our study is that among the investigated semantic similarity measures, the node-based measures performed clearly better than the edge-based measures. This finding is consistent with Pesquita *et al.* who observed that edge-based measures are not well adapted to compute the similarity among terms within GO [10]. Indeed, GO terms situated at the same depth are not necessarily similarly specific and a given distance based on the count of edges between two terms does not attest the same semantic distance for another pair of GO terms. It can also be noted that LC is an edge-based measure that provides the worst results. Thus, the use of LCA by both Ganesan and PS appears to be effective in improving edge-based measures. The selected hybrid measure was not successful, suggesting that the impact of the features in the node-based measures (*i.e.*, the IC_S_ and MICA for Zhou’s measure) is negligible compared with the edge-based features (*i.e.*, the shortest distance between the two terms to be compared for Zhou’s measure). Interestingly, we illustrated that node-based measures yield good results although they use different features. Indeed, DF is singular as it only relies on the number of ancestors of the two terms to be compared while the other four measures are based on the IC. Finally, measures using intrinsic (Nunivers and AIC) versus extrinsic (Lin and Resnik) ICs did not reveal major differences. To confirm that intrinsic and extrinsic ICs could be used equally, it would be interesting to compute the Nunivers measure with the IC_R_ (because it is closer to Lin and Resnik than AIC) and determine whether similar results would be found.

We chose to investigate nine semantic similarity measures that rely on an exhaustive panel of GO terms’ features. Notably, we did not consider some recent measures that use other relations than *is_a*. In particular, Wang *et al.* proposed a semantic similarity measure that considers *part_of* relations [43]. We believe that using all types of relations (*i.e.*, hierarchical and transversal) is an interesting approach and that axioms should also be considered, as described by Ferreira *et al.* [44]. Axioms can be used to express the meaning of concepts and relations between concepts within ontologies [45]. Thus, if the meaning of the GO terms was fully described (with a logical definition based on axioms), the GO terms could be better distinguished from their siblings (or other related terms). Some efforts have been recently made to enrich GO with such axioms [46, 47], opening up perspectives for proposing semantic similarity measures relying on their richness.

As previously mentioned, the annotation of biological data is still an open question in scientific fields [41, 42]. Thus, we analyzed two large gene sets and specifically focused on human data, but the interpretation of biological experiments can change with the evolution of GO according to various organisms. Thus, the observations reported by Tomczak *et al.* [41] and Haynes *et al.* [42] regarding the strong annotation bias in the GO annotations in which more than half of the annotations are related to approximately one fifth of the human genes have strongly guided the choice of the datasets. Therefore, human data present competitive advantages for using very well-studied genes that are very well annotated in addition to a larger set of poorly studied genes. As enrichment methods seem to tend to focus on the well-known subpart, it is truly relevant to analyze how the integration of less known data (with still evolving knowledge) can affect the interpretation of biological data. These findings are specifically related to the coverage of annotated genes. The smaller the coverage, the more pertinent the level of information might be. However, the little information that can be learned from the less-studied genes cannot be a part of the analysis. Then, to guide the reader, we also integrated in our pipeline the results given by the popular enrichment tool DAVID. As our study specifically focused on semantic similarity measures, we purposely chose an enrichment method that does not postprocess over-represented terms as this could be achieved by using Revigo [6]. Using the results provided by DAVID, one can observe a notable disadvantage in terms of the gene coverage. Obviously, individual terms have to be significantly over-represented before being analyzed by Revigo. The *a posteriori* use of semantic similarity measures cannot address this issue, and the genes exclusively annotated by sibling terms of over-represented terms are likely not considered by Revigo, which might decrease the importance of a biological function (given by the coverage that is reduced in such a case).

## 6 Conclusion

The major issues in finding gene signatures are related to investigating the biological function of gene sets. These goals can be easily achieved using enrichment methods, such as DAVID. However, there is a risk of focusing on the top of the iceberg, which contains the well-known genes. Thus, delineating the biological process mechanisms underlying a set of genes poses a great challenge in considering the whole knowledge given by data sources. An additional problem inherent to this issue is the redundant annotation information for which integration remains a largely manual process. To address these issues, bioinformatics offers various strategies ranging from enrichment analyses to semantic similarity measures. The latter approaches have been intensively studied by the scientific community to provide a large range of measures. While these measures are often combined with enrichment methods, their *a priori* use may widely impact the interpretation of biological datasets. To investigate these challenges, we developed a large-scale approach that uses semantic similarity measures within a robust interpretive analytic framework. We chose to use a straightforward set of nine measures covering various features and explored their pitfalls by examining criteria that may be good markers providing relevant information to domain experts. Thus, we analyzed the semantic similarity measures in terms of their capacity to synthesize information and provide the best trade-off for retaining detailed information.

Therefore, the main finding was that using Gene Ontology to annotate gene sets, we obtained better results with the node-based measures that used the terms’ characteristics than with measures based on edges that link these terms. Moreover, by more deeply investigating the annotation of the gene sets provided by the node-based measures, the experiments did not detect any major differences, although these measures used different features. Finally, another interesting finding that emerged from the analysis of the human modules is that enrichment methods seem to mainly focus on the well-known subpart of genes. Consequently, methods that use semantic similarity measures can help improve gene set analyses by integrating evolving knowledge (including less-studied genes).

## Supporting information

S1 Fig Percentage of covered genes of Chaussabel and Baldwin’s modules. (TIFF)

S2 Fig Percentage of covered genes of the BTM. (TIFF)
