# Supplementary figures and images for: A new method for evaluating the impacts of semantic similarity measures on the annotation of gene sets
Source: PLoS One. 2018 Nov 27;13(11):e0208037. doi: 10.1371/journal.pone.0208037 (PMC6258551; doi:10.1371/journal.pone.0208037)

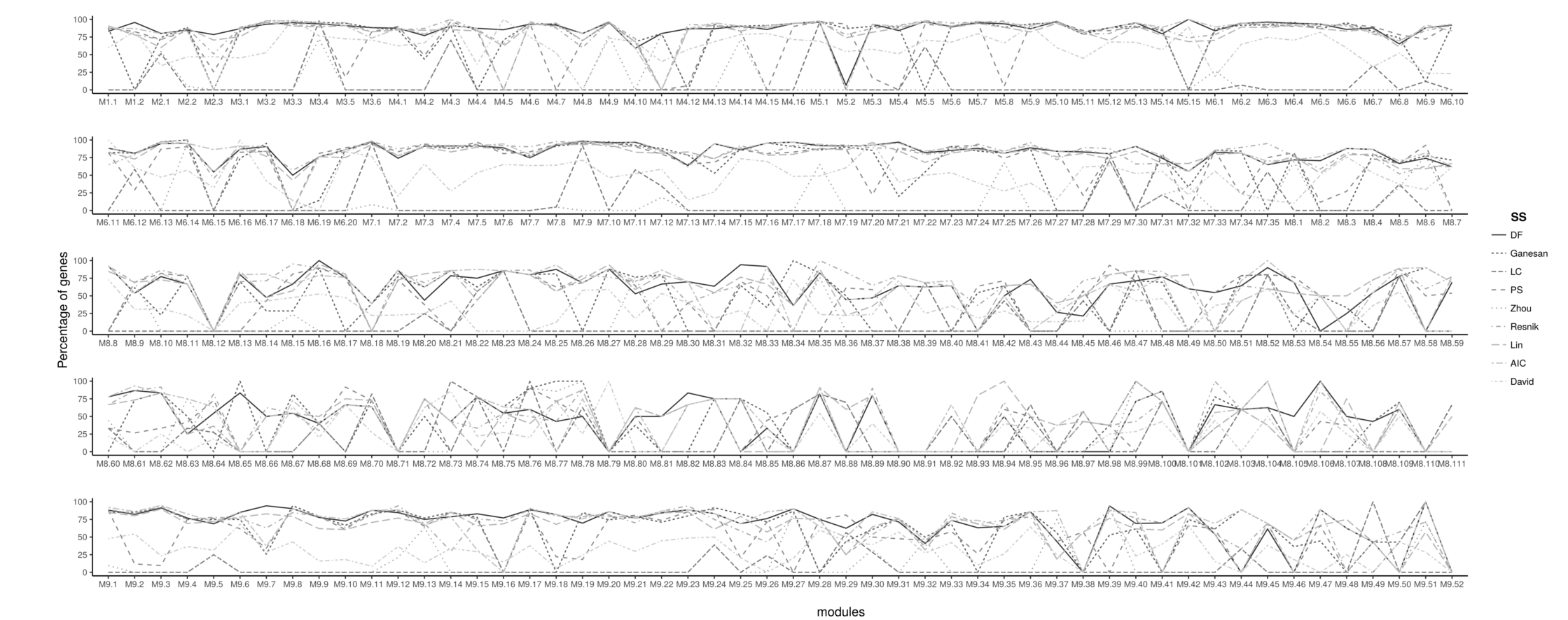

Supplement: S1 Fig — (TIFF) [file pone.0208037.s001.tiff]

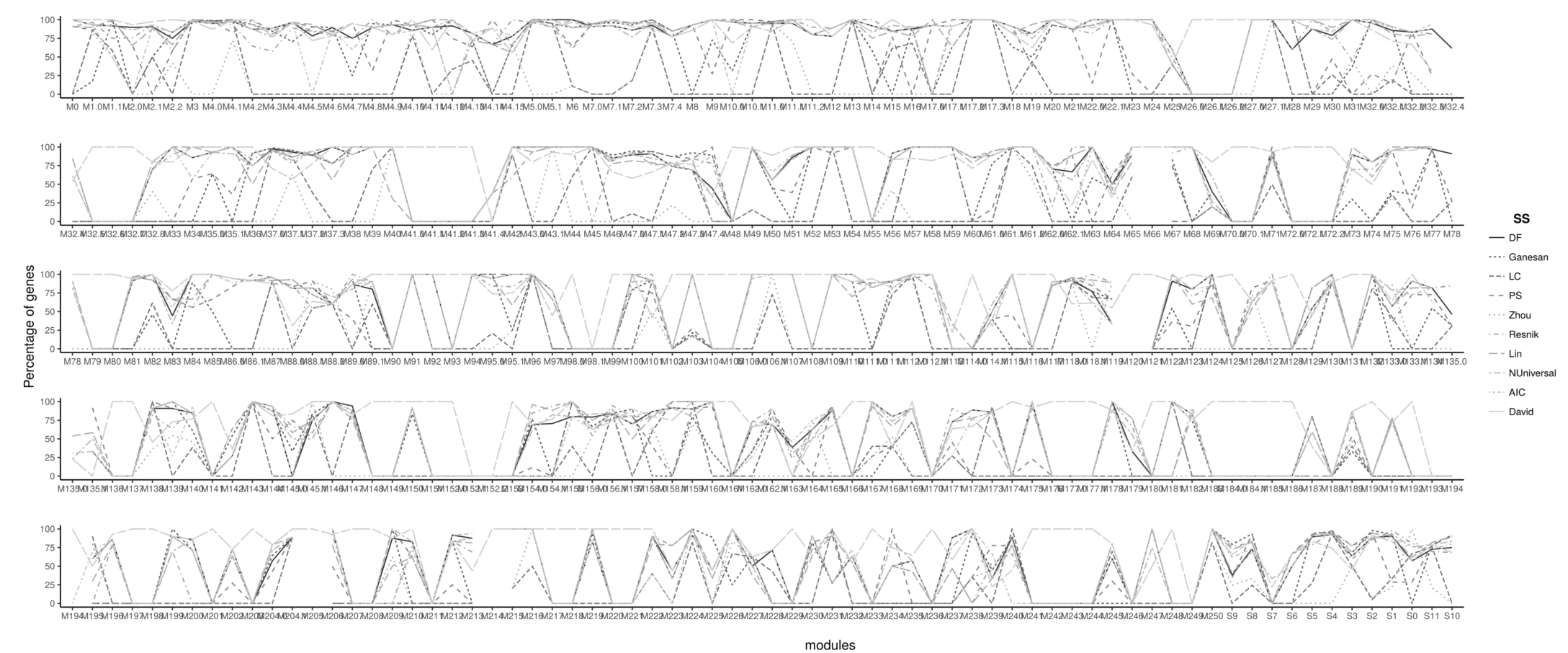

Supplement: S2 Fig — (TIFF) [file pone.0208037.s002.tiff]
